# Case Image: Mucinous Cystadenoma With Luteal Cell Proliferation During Early Pregnancy

**DOI:** 10.1002/ccr3.71565

**Published:** 2025-12-17

**Authors:** Batool Zahra, Saima Gulzar, Faiza Fatima, Ahila Ali, Hamna Ali, Aymar Akilimali

**Affiliations:** ^1^ Department of Pathology Pak Red Crescent Medical and Dental College Multan Pakistan; ^2^ Department of Gynae and Obstetrics Services Institute of Medical Sciences Lahore Pakistan; ^3^ Department of Gynae and Obstetrics Dow Medical College Karachi Pakistan; ^4^ Medical Research Circle (MedReC) Goma DR Congo

**Keywords:** luteal cell proliferation, mucinous cystadenoma, ovarian cyst, pregnancy luteoma

## Abstract

Pregnancy‐related luteal stromal proliferation can mimic ovarian neoplasia; recognizing this benign change prevents misdiagnosis and unnecessary treatment.

A 32‐year‐old woman at 12 weeks' gestation presented with right lower abdominal pain. Pelvic ultrasound revealed a simple unilocular right ovarian cyst (4.5 × 3.5 × 3 cm) adjacent to the intrauterine pregnancy (Figure 1). Laparoscopic cystectomy was performed without complications. The cyst contained scant gelatinous fluid. Histopathology demonstrated a benign mucinous cystadenoma lined by uniform mucinous epithelium. Notably, the cyst wall stroma contained abundant luteal (corpus luteum‐type) cells arranged in sheets (Figures 2, 3, 4). Mild chronic inflammation and hemosiderin‐laden macrophages were also present. The final diagnosis was mucinous cystadenoma with luteal cell proliferation. The patient recovered uneventfully and the pregnancy continued normally.

**FIGURE 1:**
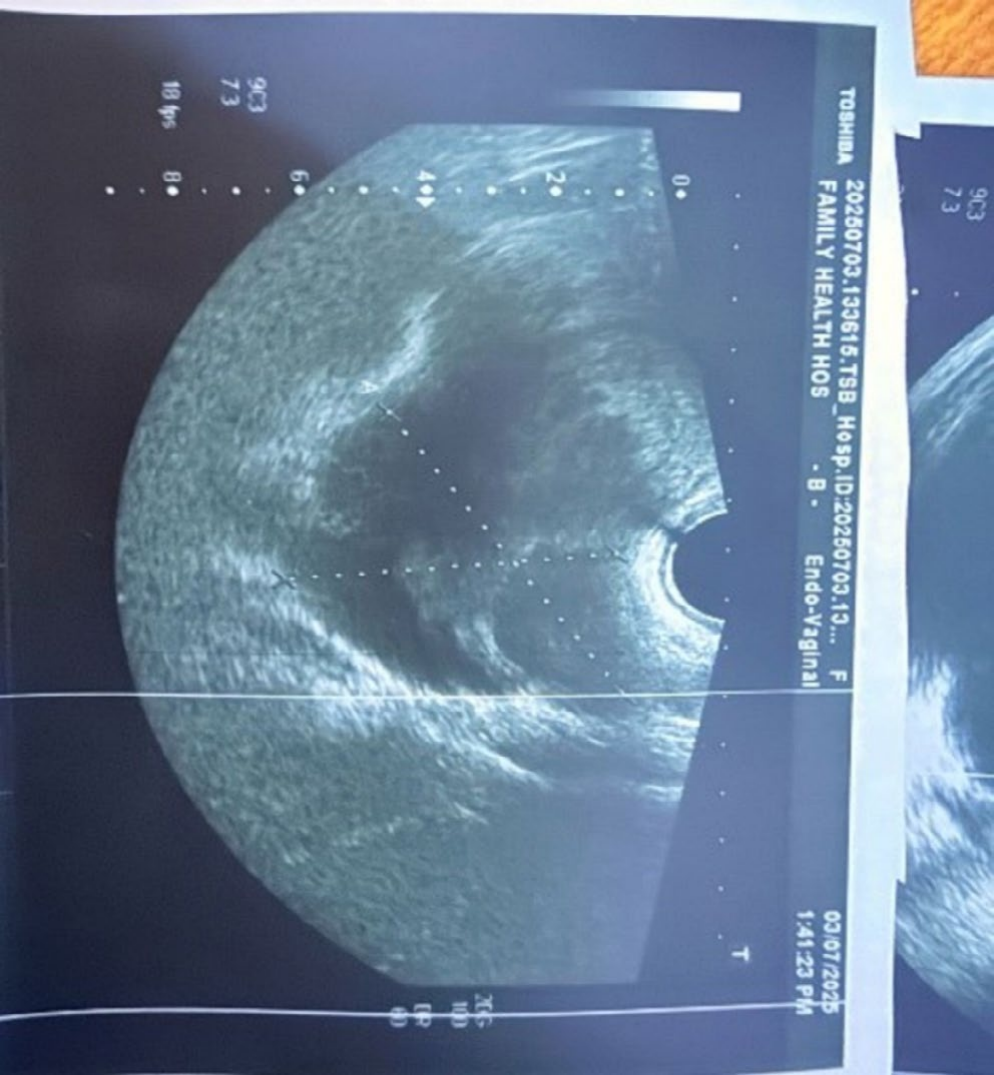
Transvaginal pelvic ultrasound image at 12 weeks' gestation, showing a simple unilocular right ovarian cyst (arrow) adjacent to the intrauterine gestational sac.

**FIGURE 2:**
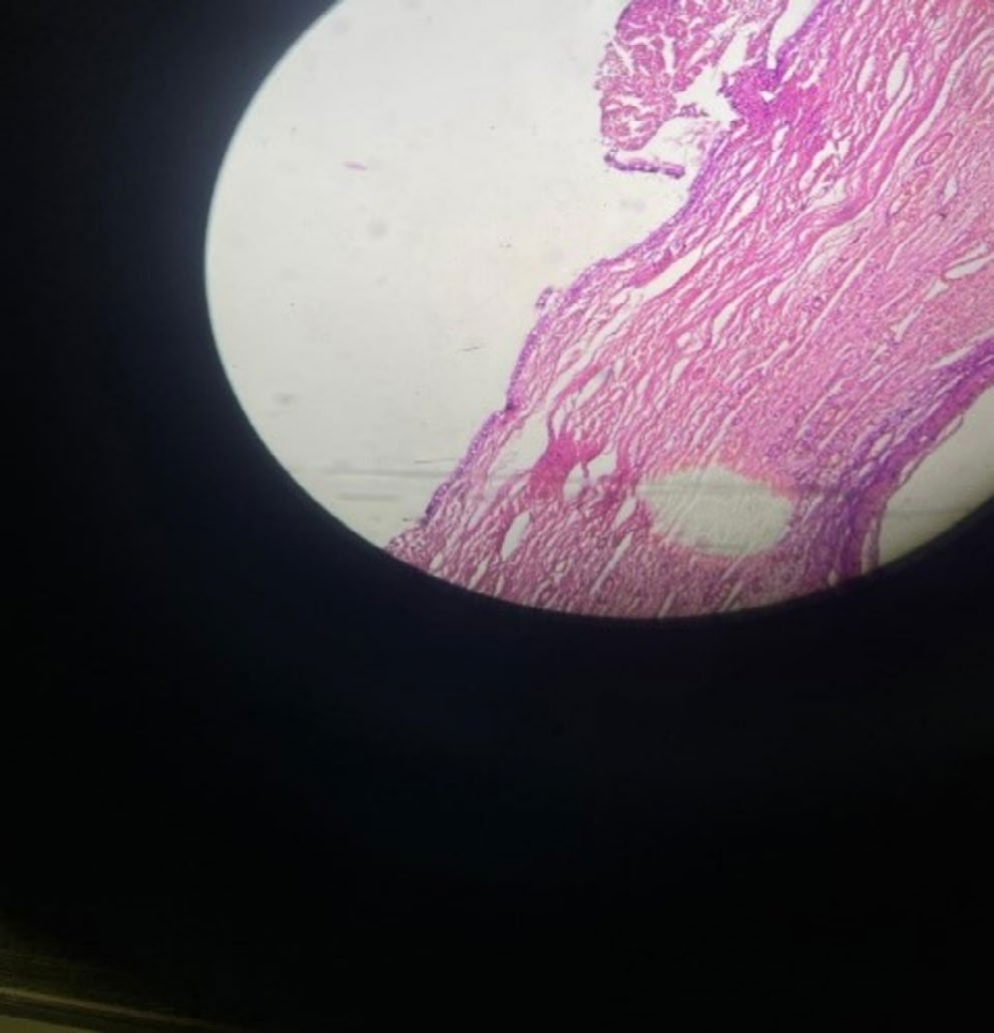
Hematoxylin and eosin‐stained section of the cyst wall (original magnification ×100) showing uniform mucinous epithelium (asterisk) and underlying fibrocollagenous stroma containing sheets of luteal (corpus luteum‐type) cells.

**FIGURE 3:**
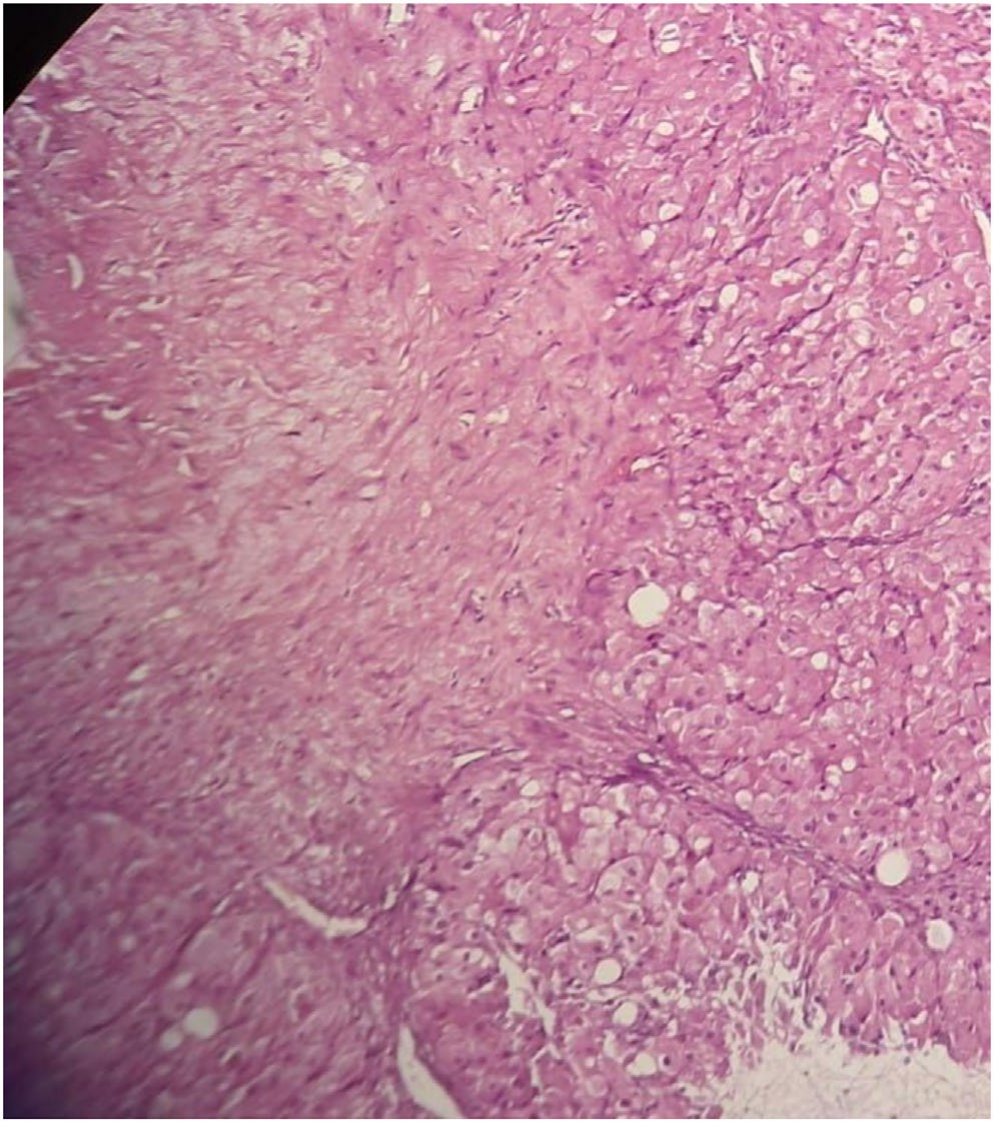
High‐power H&E view of the cyst wall (original magnification ×400) focusing on luteal cells with abundant eosinophilic granular cytoplasm and uniform round nuclei. No mitotic figures or atypia are seen.

**FIGURE 4:**
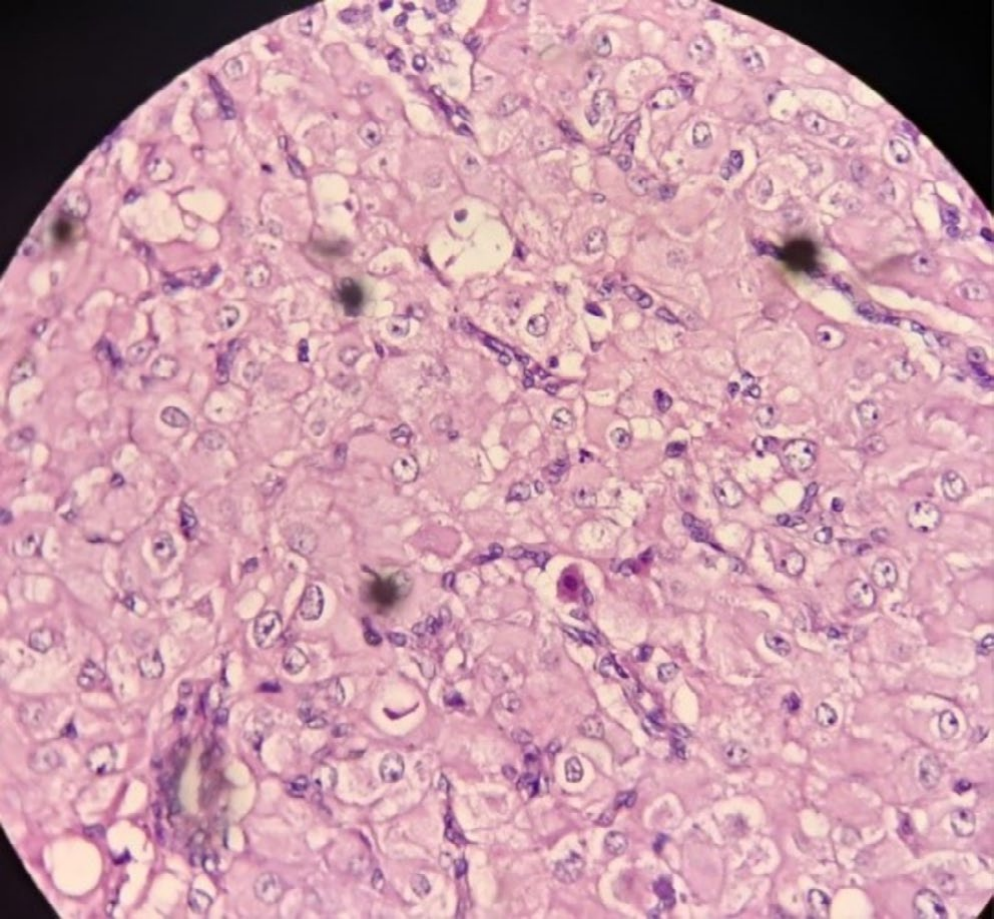
High‐power H&E view of the cyst wall (original magnification ×400) highlighting scattered hemosiderin‐laden macrophages (arrowheads) and focal chronic inflammatory cells within the luteal stroma.

Mucinous cystadenomas are among the most common ovarian epithelial tumors, and the vast majority are benign [1]. However, coexistence with marked luteal (stromal) proliferation is rare. During pregnancy, hormonal stimulation can induce luteinization of ovarian stroma (pregnancy luteoma), characterized by nests of benign luteal cells [2]. These luteal cells have abundant granular eosinophilic cytoplasm and uniform round nuclei without atypia. On microscopy (Figures 2, 3, 4), the mucinous epithelium was uniform with no dysplasia, and the luteal cells showed no mitotic activity or atypia [3]. These features favor a reactive luteal proliferation secondary to pregnancy hormones rather than a true neoplastic process.

This case highlights a diagnostic pitfall in ovarian pathology. The presence of luteal cell nests within a benign cystadenoma could mimic a neoplastic stromal tumor if unrecognized. Pathologists and clinicians should consider pregnancy‐related stromal luteinization in the differential diagnosis of ovarian masses during pregnancy. Awareness of this rare, benign association ensures accurate diagnosis and prevents unnecessary aggressive treatment.

## Author Contributions

**Batool Zahra:** conceptualization, data curation, investigation, methodology, supervision, writing – review and editing. **Saima Gulzar:** conceptualization, investigation, methodology, project administration, writing – review and editing. **Faiza Fatima:** data curation, investigation, methodology, writing – original draft. **Ahila Ali:** investigation, methodology, writing – original draft, writing – review and editing. **Hamna Ali:** data curation, methodology, writing – original draft. **Aymar Akilimali:** writing – review and editing, data curation, project administration, writing – original draft.

## Funding

The authors have nothing to report.

## Consent

Written informed consent was obtained from the patient for publication of this case and accompanying images.

## Conflicts of Interest

The authors declare no conflicts of interest.

## Data Availability

Data sharing not applicable to this article as no datasets were generated or analyzed during the current study. All relevant material is presented in the manuscript.
